# Chromosome painting in *Glyphorynchus spirurus* (Vieillot, 1819) detects a new fission in Passeriformes

**DOI:** 10.1371/journal.pone.0202040

**Published:** 2018-08-23

**Authors:** Talita Fernanda Augusto Ribas, Cleusa Yoshiko Nagamachi, Alexandre Aleixo, Melquizedec Luiz Silva Pinheiro, Patricia Caroline Mary O´Brien, Malcolm Andrew Ferguson-Smith, Fengtang Yang, Pablo Suarez, Julio Cesar Pieczarka

**Affiliations:** 1 Laboratório de Citogenética, Centro de Estudos Avançados da Biodiversidade, Instituto de Ciências Biológicas, Universidade Federal do Pará, Belém, Brazil; 2 CNPq Researcher, Conselho Nacional de Desenvolvimento Científico e Tecnológico, Brasilia, Brazil; 3 Department of Zoology, Museu Paraense Emílio Goeldi, Belém, Brazil; 4 Cambridge Resource Centre for Comparative Genomics, Department of Veterinary Medicine, University of Cambridge, Cambridge, United Kingdom; 5 Wellcome Sanger Institute, Wellcome Genome Campus, Hinxton, Cambridge, United Kingdom; 6 Instituto de Biología Subtropical (IBS), CONICET-UNaM, Puerto Iguazú, Misiones, Argentina; Fred Hutchinson Cancer Research Center, UNITED STATES

## Abstract

*Glyphorynchus spirurus* (GSP), also called the Wedge-billed Woodcreeper (Furnariidae) has an extensive distribution in the Americas, including the Atlantic coast of Brazil. Nevertheless, there is no information about its karyotype or genome organization. To contribute to the knowledge of chromosomal evolution in Passeriformes we analysed the karyotype of *Glyphorynchus spirurus* by classic and molecular cytogenetics methods. We show that *Glyphorynchus spirurus* has a 2n = 80 karyotype with a fundamental number (FN) of 84, similar to the avian putative ancestral karyotype (PAK). *Glyphorynchus spirurus* pair 1 was heteromorphic in the Tapajós population whereby the short arms varied in sizes, possibly due to a pericentric inversion, as described in other Furnariidae birds. FISH with the Histone H5 probe revealed a signal in the pericentromeric region of *G*. *spirurus* chromosome 5 and rDNA 18S showed interstitial signal in GSP-1. Chromosome painting with *Gallus gallus* (GGA) macrochromosomes 1–9 probes showed disruption of chromosome syntenies of GGA-1, 2 and 4 by fission in *Glyphorynchus spirurus*. Our results confirm that the GGA1 centric fission is a synapomorphic character for the phylogenetic branch composed of Strigiformes, Passeriformes, Columbiformes and Falconiformes. On the other hand, the GGA-2 fission is reported here for the first time in Passeriformes. Chromosome painting with BOE whole chromosome probes confirmed these rearrangements in *Glyphorynchus spirurus* revealed by *Gallus gallus* 1–9 probes, in addition to enabling the establishment of genome-wide homology map.

## Introduction

*Glyphorynchus spirurus* (wedge-billed Woodcreeper) belongs to Furnariidae, with a wide distribution in the Americas, ranging from Central America, west to the Andes, throughout central Amazonia, and south along the Atlantic coast of Brazil [1]. This species is common, occurring in different types of lowland habitats, such as *terra firme* forests and seasonally flooded lowland forests, like *várzea* and *igapó* [2].

*Glyphorynchu****s*** *spiruru*s is a polytypic taxon with thirteen subspecies [1], mostly endemic or associated with particular areas [3, 4]. Recently, high levels of genetic differentiation were found in different populations [5], suggesting the existence of several cryptic unnamed taxa.

Only two species of the Furnariidae family have had their karyotypes studied (*Sittasomus griseicapillus*, SGR and *Lepidocolaptes angustirostri*, LAN, both with 2n = 82 chromosomes), and no information on the *Glyphorynchus spirurus* karyotype can be found. Whole chromosome probes from *Gallus gallus* have been widely used in comparative bird studies, although conservation of the syntenic groups prevents the identification of intrachromosomal rearrangements [6]. *Gallus gallus* (Galliformes) has a karyotype considered less derived in respect to the putative avian ancestral karyotype. Also, Galloanserae is the sister clade to Neoaves including *Burhinus* (Charadriiformes) and *Glyphorynchus spirurus* (Passeriformes). While several species of the Oscines suborder have been analysed by chromosome painting [7–13], only one species in Suboscines (6), *Elaenia spectabili*s (Tyrannidae), has been studied so far, In Furnariidae, *Glyphorynchus spirurus* has a sister relationship to the ‘‘strong- billed” clade [14].

Although Passeriformes usually have a stable diploid number of 2n = 80, with similar macrochromosomes, chromosome painting with whole chromosome probes of *Leucopternis albicollis* has shown a complex pattern of pericentric and paracentric inversions, in this group, involving chromosomes homologous to GGA-1q, both in Oscines and Suboscines [11–12, 15]. Despite the conserved karyotype of these birds, *in silico* analyses from genome sequencing demonstrates that many intrachromosomal rearrangements, such as micro inversions, fusions and fissions have occurred in their genomes [16, 17]. Also, whole chromosome probes of *Burhinus oedicnemus* (BOE, 2n = 42) [18], a species with the lowest known diploid number among birds, have been used in association with GGA probes to identify evolutionary rearrangements in other neoaves species [18–20].

Here, we analysed for the first time the karyotype of *Glyphorynchus spirurus* with *Gallus gallus* and *B*. *oedicnemus* whole chromosome probes and also rDNA 18S and Histone H5 probes with the aim to understand the genomic organization and chromosomal evolution in birds. The results provide new information on the phylogenetic relationships in Furnariidae and Passeriformes.

## Material and methods

### Samples and chromosomal preparation

Five specimens of *Glyphorynchus spirurus* were collected from natural populations of the Brazilian Amazon in Flona Nacional do Tapajós in Belterra (2°24'05''S/55°04'40''W), (one specimen female and three males) and Santa Bárbara, Pará, Brazil (Tapajós and Belém endemic areas) (1°12'14"S/48°17'39"W), (1 male and 1 female). Bone marrow preparations were performed after Colchicine treatment [21], with modifications. Voucher specimens were deposited in the bird collection of the Museu Paraense Emilio Goeldi. JCP has a permanent field permit number 13248 from “Instituto Chico Mendes de Conservação da Biodiversidade”. The Cytogenetics Laboratory from Universidade Federal do Pará has a special permit number 19/2003 from the Brazilian Ministry of Environment for sample transport and 52/2003 for using the samples for research. The Ethics Committee (Comitê de Ética Animal da Universidade Federal do Pará) approved this research (Permit 68/2015). The specimens were maintained in the lab with food and water, free from stress, until euthanised by intraperitoneal injection of barbiturates under local anaesthesia.

### Fluorescence *in Situ* Hybridization (FISH)

Genomic DNA was extracted from a chromosome preparation of *Glyphorynchus spirurus* (Furnariidae-Passeriformes), with DNAzol [22]. Primers were designed using Pick primer software of the NCBI platform, from a mRNA partial sequence for Histone H5 fro*m Manacus vitelinicu*s (Pipridae-Passeriformes), with the sequences H5F 5’- CTACAAGGTGGGCCAGAACG and H5R 5’- TCGTAGATGAGCCCCGAGAT. Probes of Histone H5 and 18S rDNA (*Prochilodus argenteus*) were labelled with digoxigenin or biotin by PCR and FISH experiments were carried out following the procedure previously described [23].

Chromosome painting was performed with GGA (Chromosomes 1–9) and BOE whole chromosome probes according to [18]. Both probe kits were produced from chromosomes isolated by flow cytometry at the Cambridge Resource Centre for Comparative Genomics, Department of Veterinary Medicine, University of Cambridge, UK. Primary DOP-PCR products of whole sorted chromosomes were labelled either with biotin-16-dUTP (Boehringer Mannheim), fluorescein isothiocyanate-12-dUTP (Amersham), or Cy3-dUTP by taking 1μl of product to a second round of DOP-PCR using the same primer. The biotin probes were detected with avidin-Cy3 or avidin-FITC.

## Results

**Fig 1:**
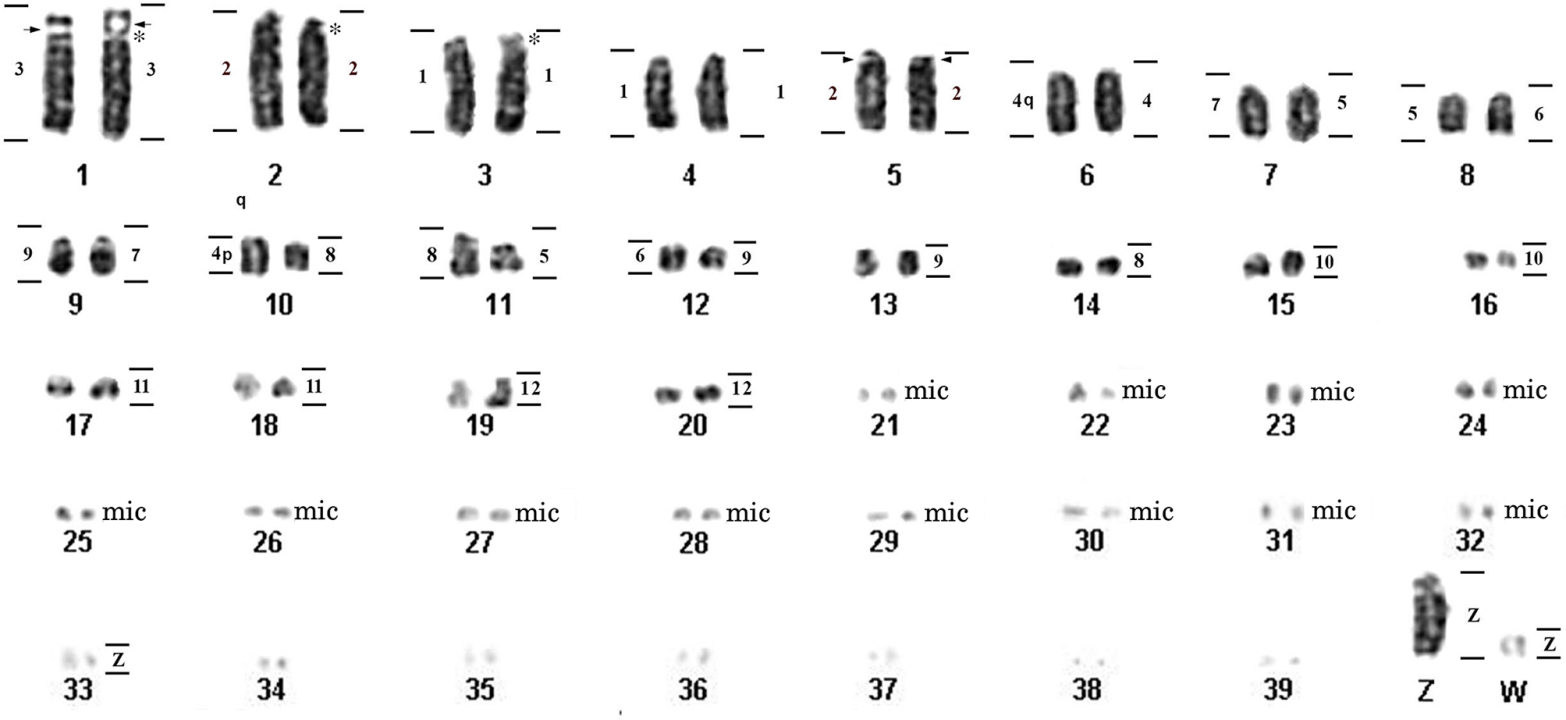
**DAPI-inverted karyotype of *Glyphorynchus spirurus* showing the localization of the corresponding chromosome probes of *Gallus gallus* (left) and *Burhinus oedicnemus* (right).** Arrows (pair 1) represent rDNA 18S gene and arrowhead (pair 5) represents histone H5 mapping. Asterisks indicate the centromeres of the subtelocentric chromosomes. Note that an unambiguous identification and ordering of microchromosomes, especially chromosomes 21–39, is beyond the scope of this paper due to the lack of reliable markers.

**Fig 2:**
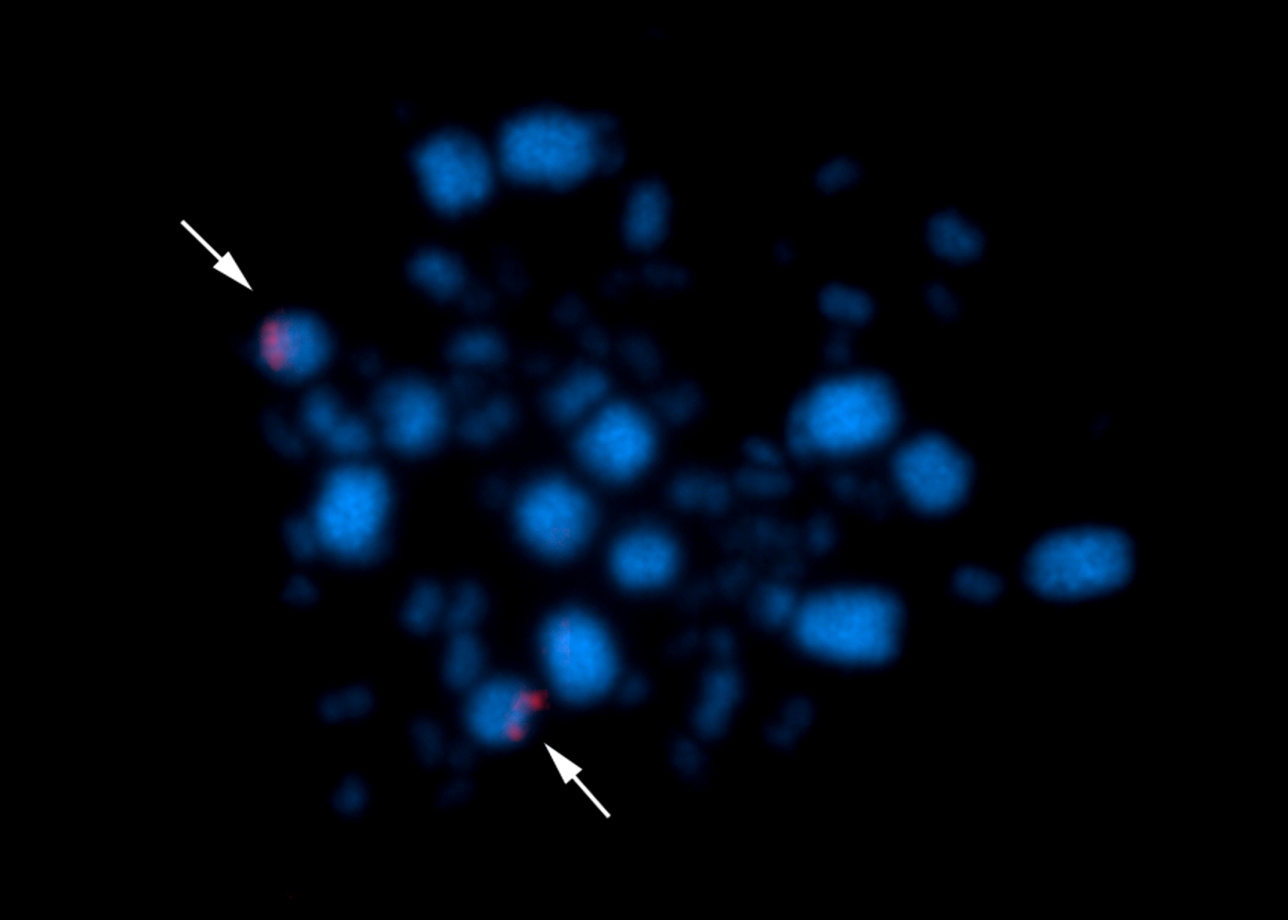
Physical mapping of Histone H5 sequences in *Glyphorynchus spirurus*. Staining with DAPI and CY3. Arrows indicate the gene location on chromosome pair 5.

**Fig 3:**
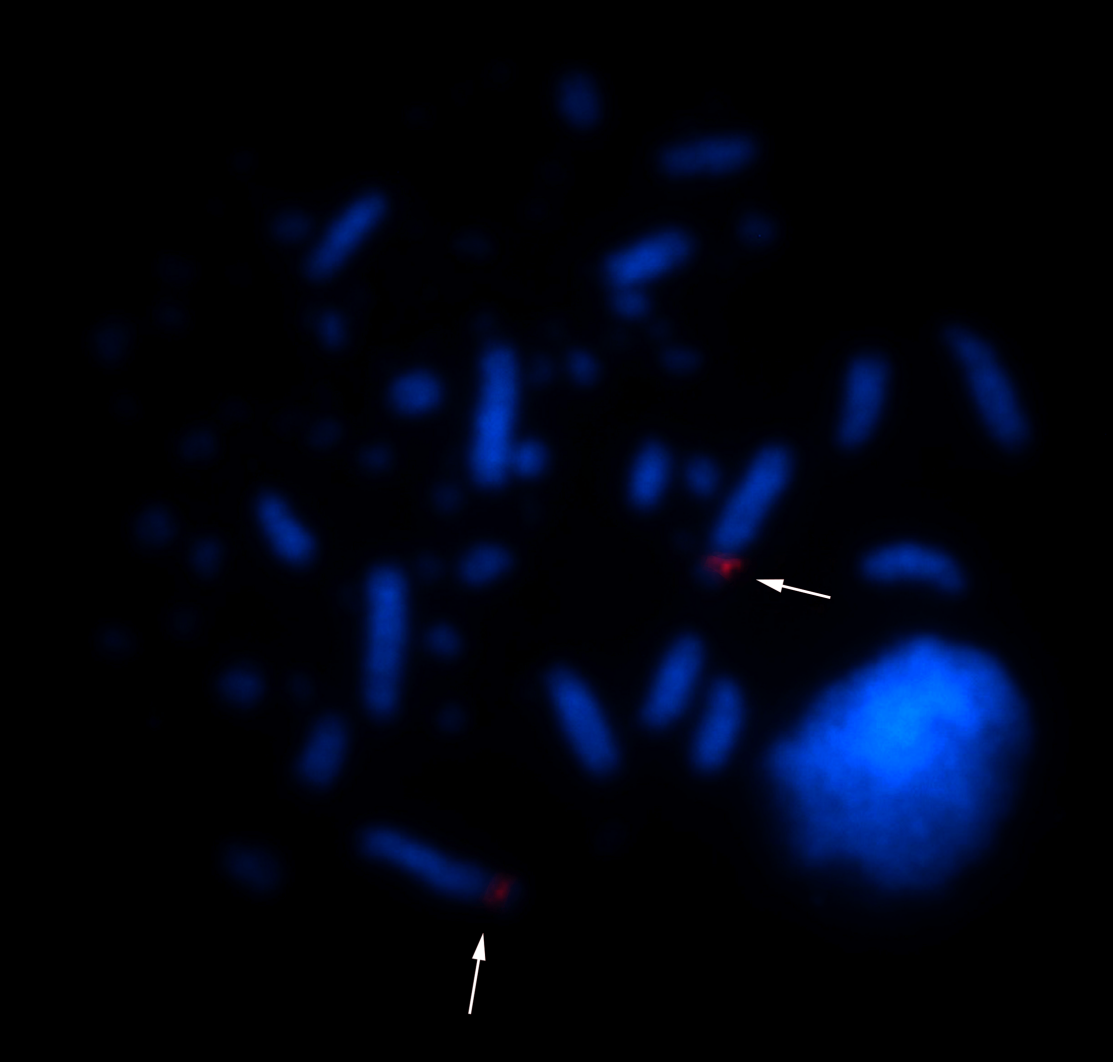
Physical mapping of rDNA 18S sequences in *Glyphorynchus spirurus* visualized with Cy3-avidin (red) and/or FITC-avidin (green). Chromosomes were stained with DAPI (blue). Arrows indicate the NOR on chromosome pair 1.

*Glyphorynchus spirurus* presented a karyotype with 2n = 80, NF = 84. The karyotype has three subtelocentric pairs, eight acrocentric pairs (macrochromosomes) and 28 pairs of microchromosomes. The Z and W chromosomes are both acrocentric (Fig 1). Chromosome pair 1 in samples from Tapajós (but not from Belém) shows a heteromorphism in the sizes of the short arms. FISH with the Histone H5 probe revealed a signal in the pericentromeric region of chromosome pair 5 (Fig 2). Hybridization with the 18S rDNA probe maps the NOR to an interstitial region of the short arm of *G*. *spirurus* pair 1 (Fig 3).

**Fig 4:**
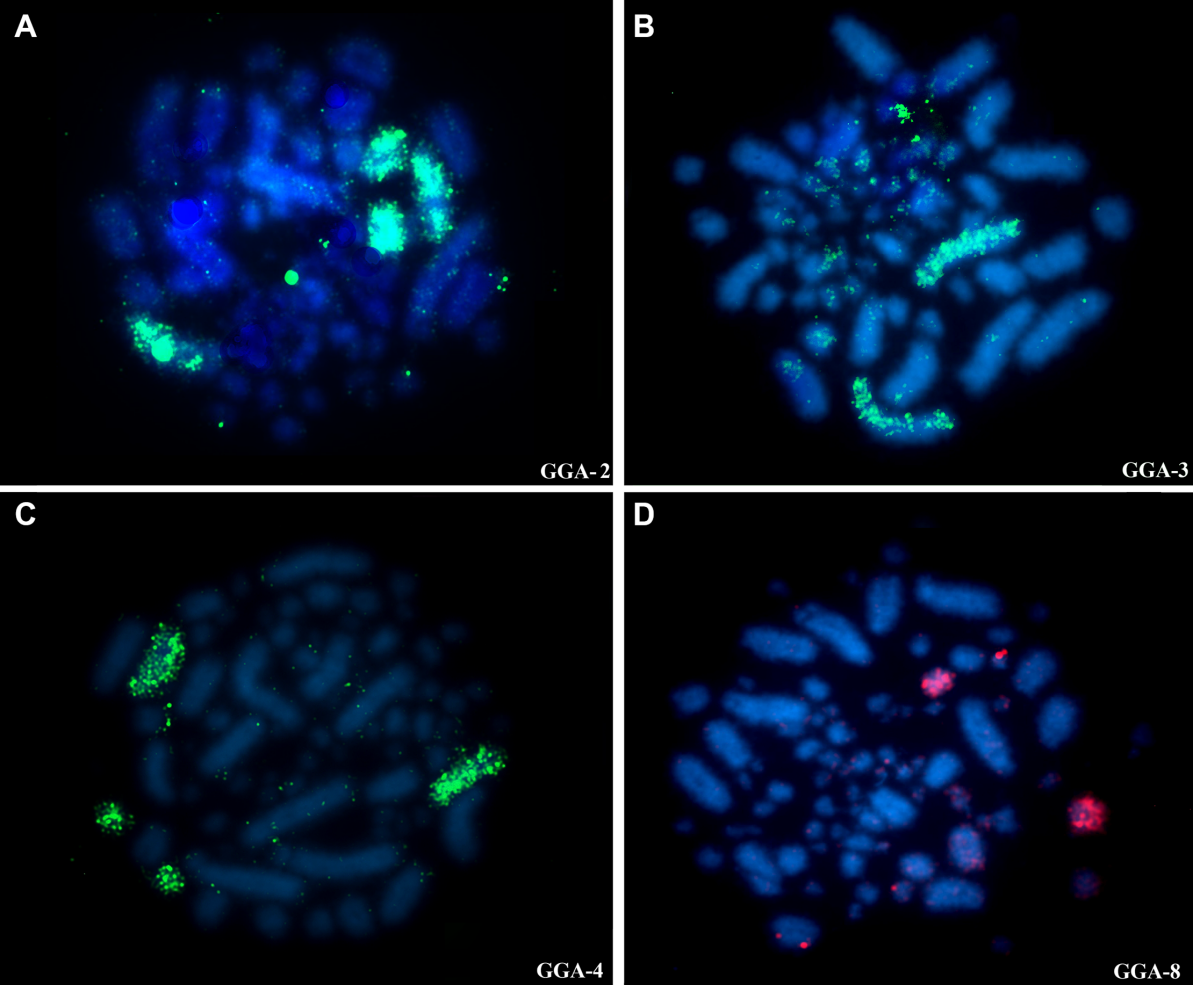
Chromosome painting with *Gallus gallus* whole chromosome probes to *Glyphorynchus spirurus*. A) GGA-2 (pairs 2, 5 and W); B) GGA-3 (pair 1); C) GGA-4 (pairs 6 and 10) and D) GGA-8 (pair 11 and W). Probes are visualized with avidin-Cy3 (red) and or avidin-FITC (green). Chromatin is stained with DAPI (blue).

**Fig 5:**
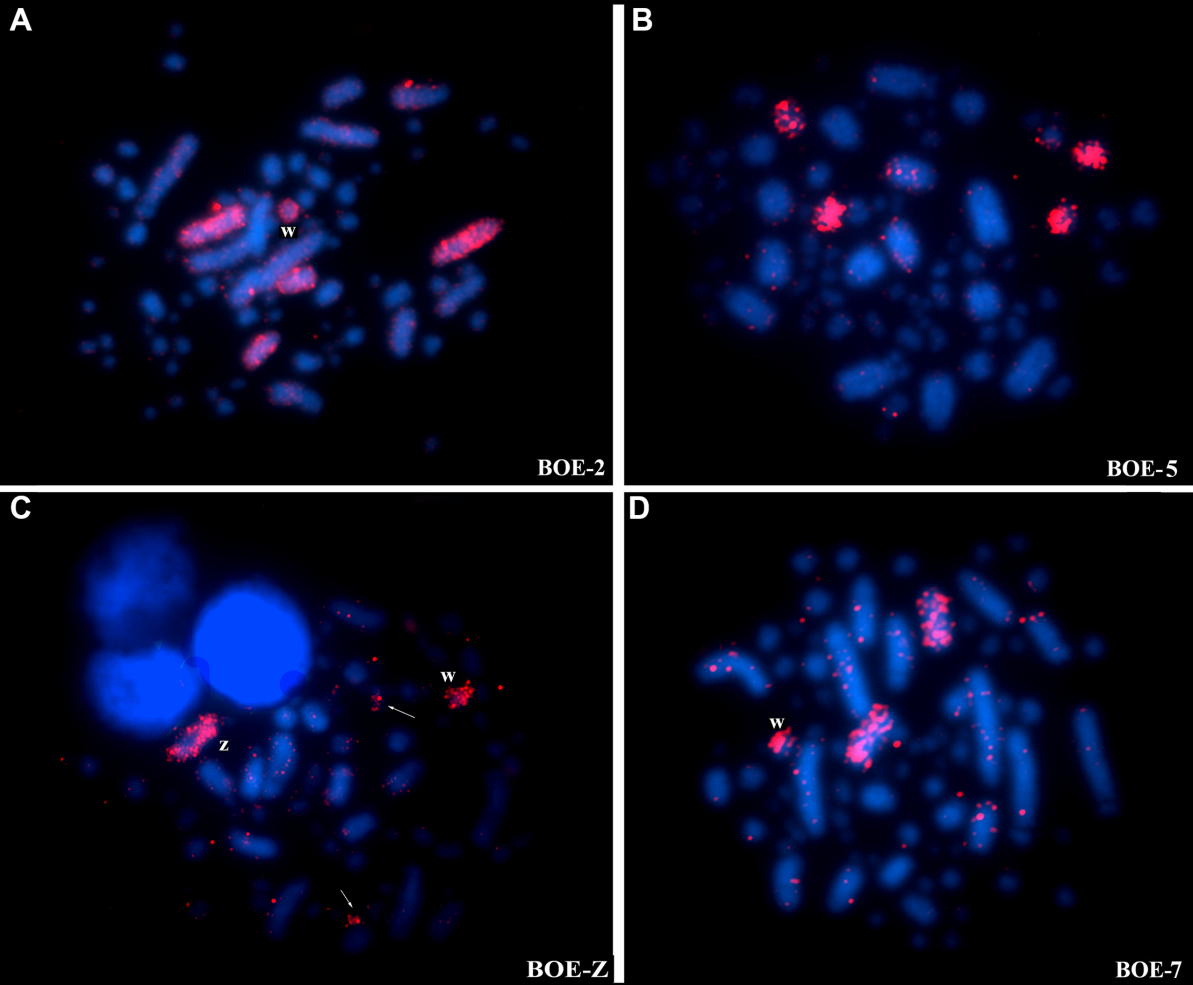
Chromosome painting with *Burhinus oedicnemus* whole chromosome probes to *Glyphorynchus spirurus*. A) BOE-2 (pairs 2 and 5); B) BOE-14 (pairs 7 and 11); C) BOE-Z (Chromosome Z, W and one michrocromosome) and D) BOE-6 (pair 9). The nomenclature to BOE probes follows Nie et al. [17]. Probes are visualized with avidin-Cy3 (red). Chromatin is stained with DAPI (blue). Note that the assignment of BOE probes to microchromosome remains highly tentative due to the difficulty in identification of *Glyphorynchus spirurus* microchromosomes.

Hybridization *of Gallus gallus* (Chromosomes 1–9) whole chromosome probes reveals 12 homologous segments on the *Glyphorynchus spirurus* genome (Figs 1 and 4) and hybridization of BOE whole chromosome probes reveals 36 homologous segments on the *Glyphorynchus spirurus* chromosomes (Figs 1 and 5, S1 and S2 Figs). The correspondence between the BOE, *Gallus gallus* and the *Glyphorynchus spirurus* karyotypes are showed in Table 1.

**Table 1:** Chromosomal correspondence between *Burhinus oedicnemus*, *Gallus gallus* and *Glyphorynchus spirurus* revealed by FISH with BOE chromosome-specific paints. Note that the chromosomal correspondence between the BOE and GGA was based on Nie et al. [17].

Chromosome		
*Burhinus oedicnemus*	*Gallus gallus*	*Glyphorynchus spirurus*
1	1	3, 4
2	2	2, 5, W
3	3	1
4	4q	6
5	7, 8	7, 11
6	5	8, W
7	9, 2 micros	9
8	4p, 1 micro	2 micros
9	2 micros	2 micros
10	2 micros	2 micros
11	2 micros	2 micros
12	2 micros	2 micros
13	2 micros	2 micros
14	2 micros	2 micros
15+16	3 micros	2 micros
17+18+19+20	1 micro	3 micros
Z	Z	Z, W, 1 micro

Micro(s) = microchromosome(s).

## Discussion

Here we describe for the first time the karyotype of *Glyphorynchus spirurus* (*sensu* [5]). The 2n = 80, karyotype is common in Passeriformes and similar to the putative bird ancestral karyotype (PAK) (2n = 80) [24], but differs by the presence of pericentric inversions in the first three pairs. This karyotype differs from that of two other Furnariidae species, *S*. *griseicapillus* and *L*. *angustirostri*, both with 2n = 82 [25] and similar karyotypes that differ from *Glyphorynchus spirurus* by structural changes in macrochromosomes (probably inversions), and by having additional microchromosomes resulting from fissions in *Glyphorynchus spirurus*.

We observed a heteromorphism in *Glyphorynchus spirurus* pair 1, where the short arm of one homologue is larger than the other in Tapajós samples when compared to samples from Belém. Since the BOE-3 and GGA-3 probes hybridize to the whole chromosome 1 in *Glyphorynchus spirurus*, this may be due to a pericentric inversion, as proposed by [25] for SGR and LAN. The Belém and Tapajós populations form distinct, yet closely related clades in a recently published phylogeography of *G*. *spirurus*, and are separated by an uncorrected genetic distance of 1.6% in the cytochrome *b* mitochondrial gene [5]. Although closely related, this degree of genetic divergence is consistent with the important chromosome differences between these populations documented herein and, in fact, indicate that some of the cryptic diversity uncovered in *Glyphorynchus* and other Suboscines passerines may be related to chromosomal differences. Therefore, we predict that even more striking chromosomal differences will be found amongst more distantly related and genetically divergent populations of *G*. *spirurus*, such as between the Guiana shield population in northeastern Amazonia and the remaining populations, which are separated by an uncorrected genetic of over 5% in the cytochrome *b* gene [5].

We also report the physical mapping of the H5 histone gene for the first time in birds and found it tandemly repeated close to the centromere of *Glyphorynchus spirurus* chromosome five (Fig 2). This histone has an important role on regulation and physiology since it partially replaces histone H1 in mature erythrocytes and is exclusive to the avian genome [26]. These results open up a new perspective about its organization and localization as a cytogenetic marker in birds.

### Chromosomal rearrangements between *Gallus gallus* and *Glyphorynchus spirurus* and their occurrence in Passeriformes

**Fig 6:**
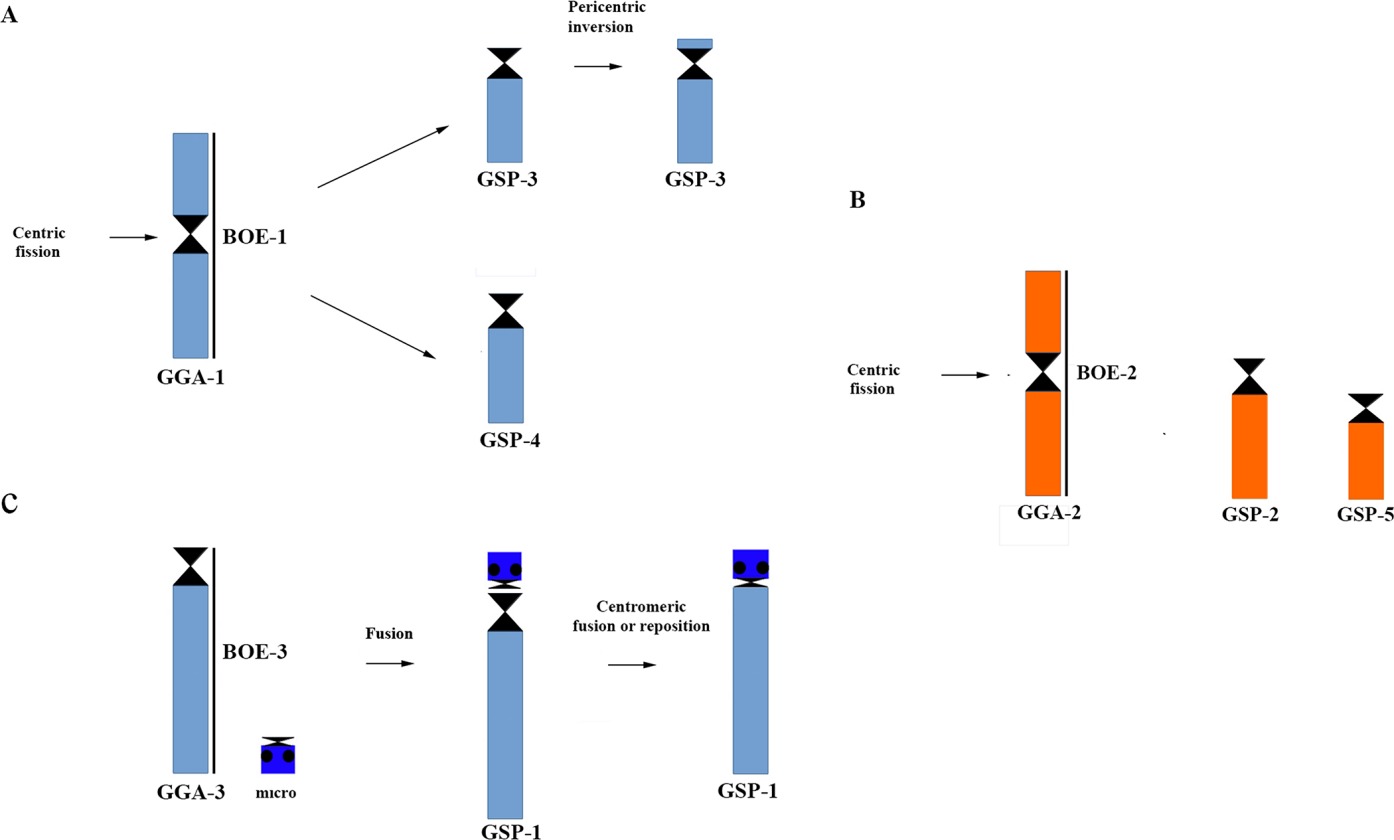
**Ideogram showing fission/fusion rearrangements to *Gallus gallus* chromosomes in *Glyphorynchus spirurus*:** A) GGA-1 fission generating two chromosomes in GSP; B) GGA-2 fission in GSP (GSP-2 and 5), and C) GGA-3 Robertsonian fusion with a microchromosome in GSP-1.

Chromosome painting with *Gallus gallus* 1–9 probes shows that majority of these GGA chromosomes were conserved in the *Glyphorynchus spirurus* karyotype, except for the fission of *Gallus gallus* 1, 2 and 4 into six *Glyphorynchus spirurus* pairs (Figs 1 and 6). The *Gallus gallus* 2 fission was described in *Buteo buteo*, *Gyps fulvus* and *Gyps himalayensis* (Accipitriformes) [19], but in these species, unlike in *Glyphorynchus spirurus*, this chromosome is fused to one microchromosome. Although the previous demonstration that the fission of GGA-2 is an old rearrangement, found in all phylogenetic branches of birds [24], it is described here for the first time in Passeriformes and may be a common trait in the Furnariidae [25]. SGR and LAN have chromosomes of similar morphology and size that are possibly homologous to *Glyphorynchus spirurus* chromosomes 2 and 5. Also, using a universal set of avian bacterial artificial chromosome (BAC) probes and chromosome painting with BOE whole chromosome probes, we have found the GGA-2 fission in four species of Thamnophilidae birds (data in preparation).

On the other hand, fission of GGA-1 is a more recent rearrangement, being restricted to the phylogenetic branch that gave rise to Strigiformes, Passeriformes, Columbiformes and Falconiformes [10, 19–20, 24, 27–28]. GGA-1 has experienced multiple fissions especially in Accipitriformes with one to six hybridization signals, four in *Gypaetus barbatus* and six in *Harpia harpia* [19, 24, 29–30].

The split of GGA-4 into two pairs is considered to be the ancestral form, so their fusion in *Gallus gallus* is apomorphic [24]. The synteny of *Gallus* macrochromosomes 1–10 in three different orders of birds, including Passeriformes was analysed [10]. We found the GGA-1 fission in all species, while GGA-4 was split in all except in blackcap and no one showed the fission in GGA-2. The *Gallus gallus* chromosome one fission has been found in Oscines (*Turdus* [11], *Saltator* [12]), Suboscines (*Elaenia* [15]) and also here in *Glyphorynchus spirurus*, and has been well accepted as a synapomorphy for Passeriformes [10, 15]. However, as this fission appears in the branch supporting Strigiformes, Passeriformes and Falconiformes [24] and thus could be a synapomorphic trait for all these orders.

Finally, we suggest that a microchromosome fusion has occurred in the *Glyphorynchus spirurus* pair 1p terminal portion (homologous to GGA-3). This may have resulted from fusion of the NOR-bearing *Gallus gallus* microchromosome 16 with *Glyphorynchus spirurus* chromosome one (Fig 6). Evidence of fusions involving GGA-3 and one small chromosome has been found previously in *Eleania* and *Turdus*, despite not involving a NOR-bearing chromosome [11, 15]. We did not find signs of this hybridization by chromosome painting, but this may be because the translocation involved only a small part of the microchromosome. Alternatively, it is possible that only the NOR had moved to GSP-1.

### Chromosomal rearrangements between *Burhinus oedicnemus* and *Glyphorynchus spirurus* and their presence/absence in non-Passeriformes

Chromosome painting with BOE probes shows that only four syntenic groups were conserved in the *Glyphorynchus spirurus* karyotype (BOE 3, 4, 6 and 7) and confirmed the split of *Gallus gallus* 1, 2 and 4, without intrachromosomal rearrangement when compared to the results with GGA probes. As BOE-7 is metacentric and GSP-9 acrocentric, we suppose that they differ by one pericentric inversion, and the acrocentric form (GSP-9) could be apomorphic because this has not been observed in other species hybridized with BOE probes [19, 20].

*Gallus gallus* and BOE whole chromosome probes were used in five species of birds belonging to five different avian orders: *Nymphicus hollandicus* (2n = 72), NHO (Psittaciformes); *Larus argentatus* (2n = 70), LAR (Charadriiformes); *Columba livia* (2n = 80), CLI (Columbiformes); *Strix nebulosa* (2n = 82), SNE (Strigiformes) and *Fulica atra* (2n = 92), FAT (Gruiformes) [20]. These species showed between 28 and 33 signals with BOE probes, while we obtained 36 signals in GSP (Fig 1), despite the considerable variation of 2n among those species, ranging from 2n = 72 in NHO, 2n = 70 in LAR to 2n = 92 in FAT.

While BOE-1, homologous to GGA-1, was conserved as one pair (LAR, CLI and FAT) or split into two pairs (NHO and NSE) as we observed in *Glyphorynchus spirurus*, BOE-2 corresponding to GGA-2 was conserved and showed variation only relative to the centromere position. Similarly BOE-1 and BOE-3 (GGA-3) were also conserved in all species studied by [20] and in *Glyphorynchus spirurus* with some variation in chromosome morphology. BOE 4, homologous to GGA-4q and BOE-8 homologous to GGA4p [18] has an interesting history among birds. The *Gallus gallus* chromosome four split into two pairs (a plesiomorphic trait), is maintained in most species including *Glyphorynchus spirurus* and is described as the most puzzling finding in avian karyotype evolution [24]. BOE-4 hybridized to one pair in GSP (GSP-6) and in all species analyzed by [20] except for FAT. BOE-8 showed two signals in NHO, CLI, SNE, FAT and GSI. On the other hand, it showed only one signal in CAR, as in BOE. Since BOE-8 is formed by GGA-4p and 1 microchromosome, the ancestral trait remains intact in these birds.

BOE chromosomes 17–20 probes produced one to four signals in all species including GSP, with some exceptions where no signal was detected in the six species [20 and present study]. In *Glyphorynchus spirurus* 34 to 39, the stone curlew probes failed to produce hybridization signals. The microchromosomes are small and it is possible that there is insufficient resolution to detect them by FISH. Another explanation is that microchromosomes have a high density of repetitive sequences, mainly telomeric [31] and commonly fail to hybridize with painting probes.

We also found in *Glyphorynchus spirurus* that the BOE-Z probe hybridized to two microchromosome pairs, the W and two BOE autosomal pairs. Apart from BOE-Z, BOE-2 and 6 also hybridized to GSP-W. These regions may have similar repetitive regions or are the result of cross hybridization as observed by [18] in *Gallus gallus* and in other species [19, 20]. The acrocentric chromosome Z in *Glyphorynchus spirurus* must have experienced an inversion as shown by comparison with BOE and *Gallus gallus*. Inversions of the Z are common in birds [32].

## Conclusions

We describe, for the first time, the chromosomal homology of *Gallus gallus* and BOE in one bird of the Furnariidae family, *Glyphorynchus spirurus*. Our results indicate that *Glyphorynchus spirurus* has a chromosomal heteromorphism in the first pair of chromosomes which also bears the NOR. The presence of the NOR in the largest chromosome is a derivative trait in birds and could be frequent in the Furnariidae family. Finally, we report for the first time the fission of GGA-2 in Passeriformes and show that the GGA-1 fission is not a synapomorphy confined only to Passeriformes birds.

## Supporting information

S1 Fig Hybridization of each *Burhinus oedicnemus* whole chromosome probe (macrochromosomes) on chromosome pairs of *Glyphorynchus spirurus*. (TIF)

S2 Fig Hybridization of each *Gallus gallus* whole chromosome probe on chromosome pairs of *Glyphorynchus spirurus*. Unfortunately, not all GGA probes worked on our sample, so in some situations we followed the findings with *Gallus gallu*s probes based on Nie et al. [18].
(TIF)
