# Supplementary figures and images for: Chromosome painting in Glyphorynchus spirurus (Vieillot, 1819) detects a new fission in Passeriformes
Source: PLoS One. 2018 Aug 23;13(8):e0202040. doi: 10.1371/journal.pone.0202040 (PMC6107148; doi:10.1371/journal.pone.0202040)

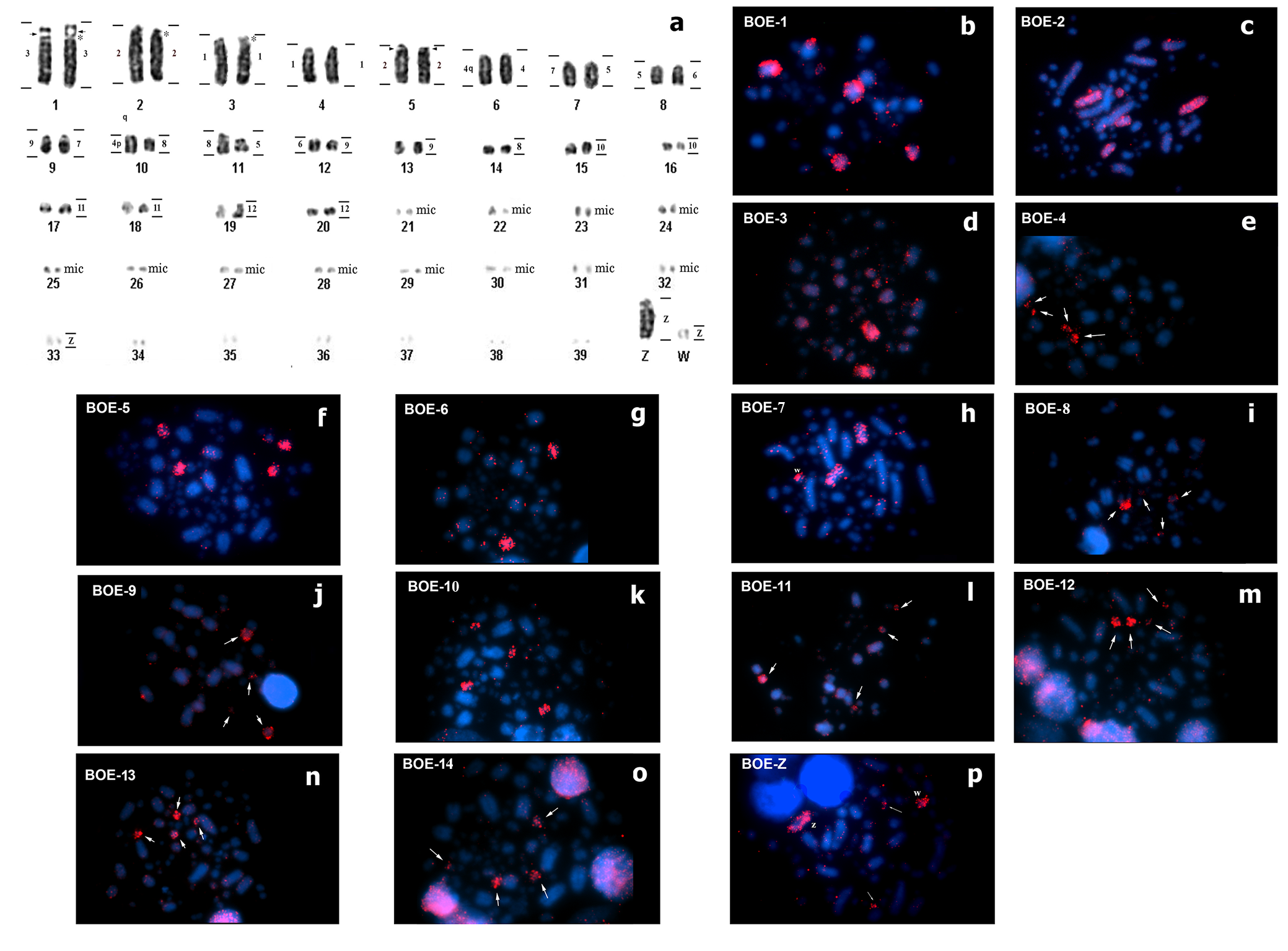

Supplement: S1 Fig — (TIF) [file pone.0202040.s001.tif]

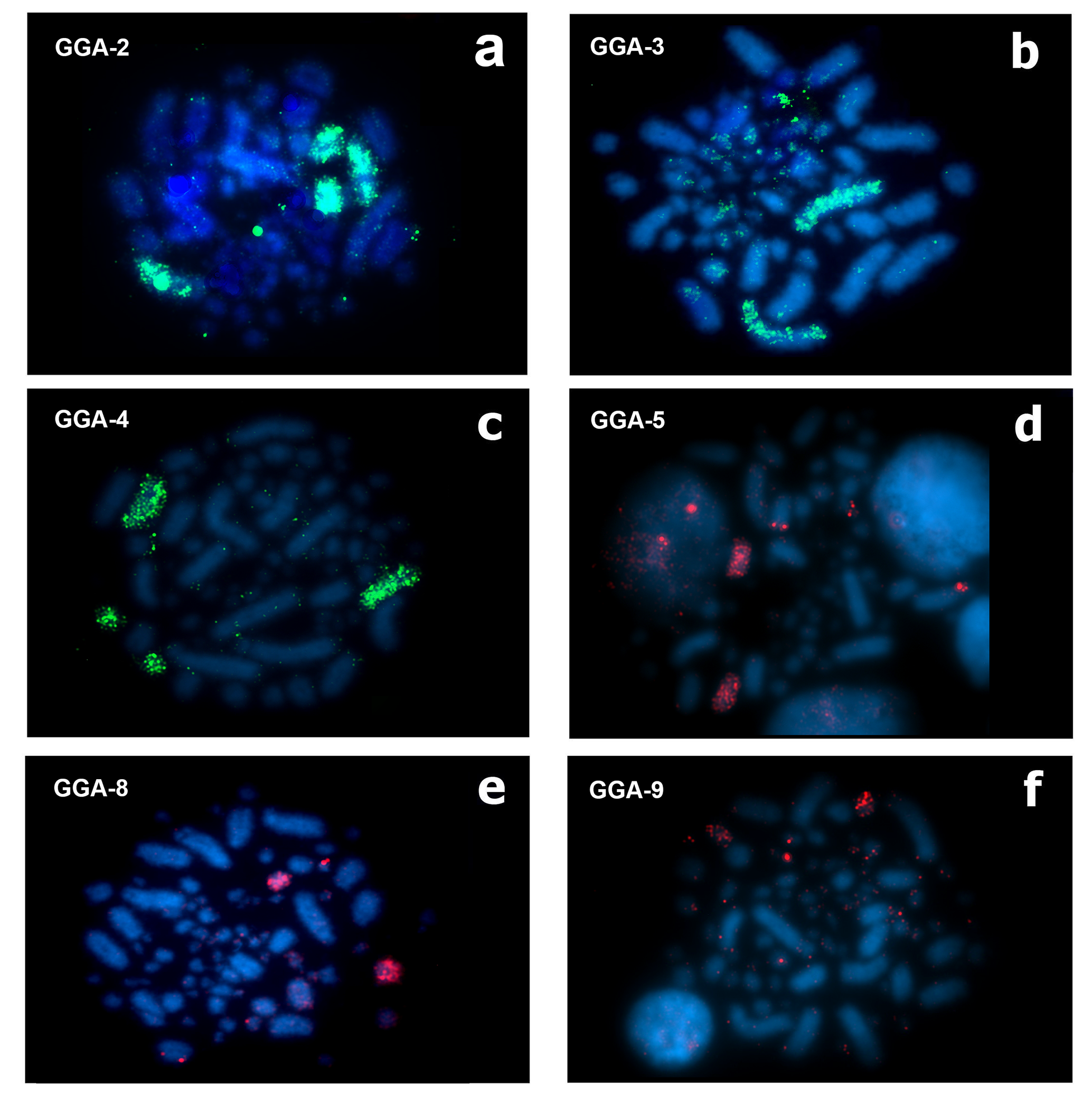

Supplement: S2 Fig — Unfortunately, not all GGA probes worked on our sample, so in some situations we followed the findings with Gallus gallus probes based on Nie et al. [18]. (TIF) [file pone.0202040.s002.tif]
